# Case Report: Prolonged survival with short-course triplet induction and dual maintenance in recurrent/metastatic cervical cancer

**DOI:** 10.3389/fonc.2026.1961384

**Published:** 2026-10-19

**Authors:** Yiqing Duan, Dan Liu, Qian Huang, Jianghan Man, Li Li, Yuanbo Xue

**Affiliations:** Department of Cancer Biotherapy Center, Yunnan Cancer Hospital, The Third Affiliated Hospital of Kunming Medical University, Peking University Cancer Hospital Yunnan, Kunming, Yunnan, China

**Keywords:** case series, cervical cancer, immune checkpoint inhibitors, immunotherapy, PD-1 inhibitor, recurrent or metastatic

## Abstract

The median overall survival (OS) for patients with recurrent or metastatic cervical cancer after progression on first-line therapy is only 5–12 months. We retrospectively reviewed four patients who received modified induction–maintenance therapy. All of the patients achieved clinical benefit to varying degrees, with one attaining progression-free survival (PFS) of over 58 months and an OS of 86 months. However, these findings should be interpreted with caution and warrant validation in larger prospective studies.

## 1 Introduction

Cervical cancer ranks as the fourth most frequently diagnosed malignancy in women worldwide (1). Prognosis is strongly correlated with the International Federation of Gynecology and Obstetrics (FIGO) stage. For stage IB disease, the 5-year overall survival (OS) is 80%–90% (2), while for locally advanced stages (IB3–IVA), it falls to approximately 74%. Even after curative-intent chemoradiation, 30%–40% of locally advanced cases eventually relapse or metastasize (3).

In recent years, immune checkpoint inhibitors in combination with chemotherapy and anti-angiogenic agents have become the new standard-of-care first-line treatment for patients with recurrent or metastatic disease. In the phase III KEYNOTE-826 study, the addition of bevacizumab to pembrolizumab plus chemotherapy (median exposure, 13.8–15.6 months) prolonged the OS in programmed death-ligand 1 (PD-L1) combined positive score (CPS) ≥1 patients to 28.6 months *versus* 16.5 months in controls (4, 5). Similarly, the COMPASSION-16 trial demonstrated that cadonilimab plus platinum-based chemotherapy (±bevacizumab) improved the median progression-free survival (PFS) to 13.3 months across the entire cohort in the updated analysis (*vs*. 8.2 months for placebo, HR = 0.62) and increased the objective response from 68% to 79% (7). The phase 3 GOG 240 trial further demonstrated that the addition of bevacizumab extended the OS from 13.3 to 16.8 months (HR = 0.77, 95%CI = 0.62–0.95) (6). In the innovaTV 205 trial, tisotumab vedotin (TV) plus pembrolizumab achieved a median OS of 15.3 months in the second-line setting, increasing to 30.7 months in PD-L1-positive patients (8, 9).

Nevertheless, these strategies have inherent drawbacks. In KEYNOTE-826, the survival benefit was largely confined to PD-L1-positive subjects, while the PD-L1-negative patients derived no meaningful OS advantage (HR = 0.91) (5). Furthermore, prolonged triple-drug administration until progression is associated with considerable toxicity and financial burden. Once the disease progresses during or after first-line therapy, treatment options become increasingly limited, particularly in patients with deteriorating clinical status.

To overcome the toxic side effects of prolonged chemotherapy and to achieve clinically meaningful survival for patients, we adopted a modified induction–maintenance therapy (MIMT): a sequential triplet-induction and dual-maintenance strategy based on optimized treatment timing. This strategy comprises short-course triplet induction with chemotherapy, an immune checkpoint inhibitor, and anti-angiogenic therapy, followed by dual maintenance after disease control is achieved. Indeed, all four patients achieved substantial survival with preserved quality of life.

## 2 Case presentation

A chronological overview of the diagnosis, treatment, and outcomes for each patient is provided in **Table 1**.

**Table 1:** Timeline of the patients.

Case	Diagnosis	Recurrence	Induction therapy (time)	Maintenance therapy (time)	PFS (months)	OS (months)	Last follow-up	Status
1	January 2020	October 2020	CTx+BEV/4 cycles (January–April 2021)	BEV+TOR/5 cycles (April–September 2021)	NE	44	September 2023	Deceased
2	February 2020	April 2021	CTx+BEV+TIS/2 cycles (May–June 2021)	BEV+TIS/10 cycles (July 2021–April 2022)	17/7.5	43	September 2023	Deceased
3	August 2019	July 2021	CTx+BEV+TOR/2 cycles (August–September 2021)	BEV+PD-1/6 cycles + intermittent (October 2021–April 2022)	46/10	>83	July 2026	Alive
4	May 2019	March 2021	CTx+BEV+TOR/2 cycles (August 2021)	BEV+TOR/ongoing (September 2021–present)	>58	86	July 2026	Alive

CTx, chemotherapy; BEV, bevacizumab; TOR, toripalimab; TIS, tislelizumab; PD-1, programmed cell death protein 1; PFS, progression-free survival; OS, overall survival; NE, not evaluable.

### 2.1 Case 1

The patient is a 53-year-old woman who was diagnosed with cervical cancer in January 2020. On February 14, she underwent total abdominal hysterectomy with bilateral salpingo-oophorectomy, with postoperative pathology confirming cervical adenocarcinoma staged as FIGO IIB. Subsequently, she received adjuvant concurrent chemoradiotherapy (the exact dose was unavailable).

In October 2020, she developed progressive abdominal distension. Imaging the following month showed large-volume ascites, peritoneal thickening, and mesenteric whirlpool-like aggregation, suggestive of peritoneal metastasis and FIGO stage IVB. The patient had an Eastern Cooperative Oncology Group (ECOG) performance status (PS) score of 2 at admission. From 3 January to 2 April 2021, the patient received albumin-bound paclitaxel (260 mg/m^2^) plus carboplatin (area under the curve, AUC = 5) and bevacizumab (15 mg/kg) for a total of four cycles. Imaging after two cycles of treatment showed stable disease (SD) based on the Response Evaluation Criteria in Solid Tumors version 1.1 (RECIST 1.1). From 27 April to 26 September 2021, the treatment was switched to bevacizumab (15 mg/kg) combined with toripalimab (240 mg) for five maintenance cycles at our institution. Imaging after two cycles of treatment showed partial response (PR) (**Figure 1**). In September 2021, cytological examination of ascitic fluid revealed no malignant cells. Thereafter, due to financial constraints, the patient did not return for regular imaging follow-up. According to information provided by the patient’s family, she died in September 2023. However, the details regarding her subsequent treatment, disease course, and the exact cause of death were unavailable, the timing of disease progression could not be determined, and PFS was not assessable. The OS, calculated from the initial diagnosis to the reported time of death, was approximately 44 months.

**Figure 1:**
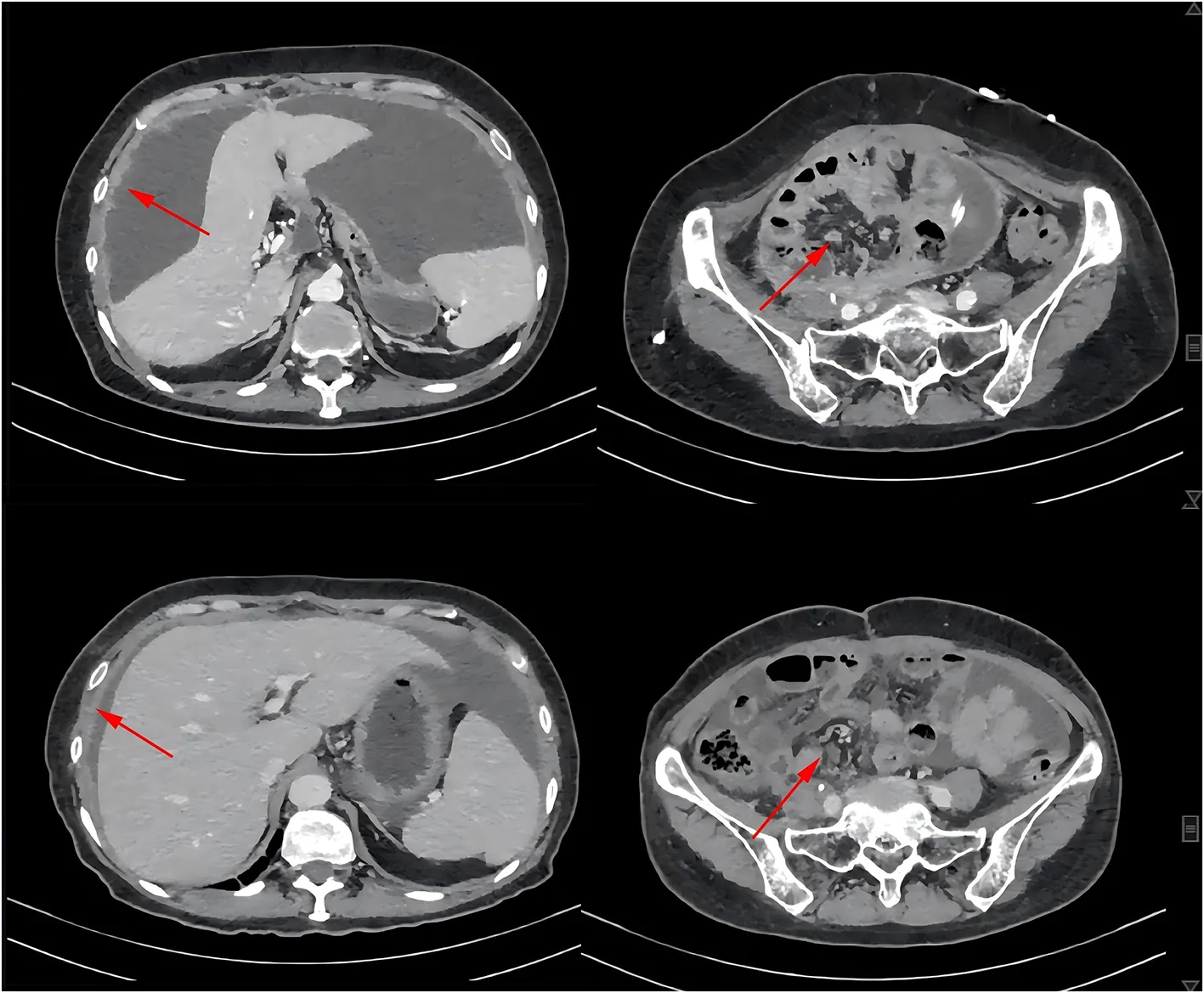
**(A)** Baseline contrast-enhanced CT scan of the upper abdomen demonstrating diffuse peritoneal thickening with multiple nodules. **(B)** Baseline CT scan of the lower abdomen and pelvis revealing massive ascites and omental stranding. **(C)** Follow-up CT scan at the corresponding level to **(A)**, after the therapy, showing marked regression of the peritoneal lesions and reduction in nodule size. **(D)** Follow-up CT scan at the corresponding level to **(B)**, acquired at the same time as **(C)**, showing a significant decrease in ascites. Images **(A, B)** were taken at baseline, while images **(C)** and **(D)** were taken after treatment. Red arrows indicate the representative peritoneal lesions and ascites.

### 2.2 Case 2

The patient is a 53-year-old woman who was diagnosed with FIGO stage IIIC1 poorly to moderately differentiated squamous cell carcinoma of the cervix on 27 February 2020. On admission, her ECOG PS score was 1. She subsequently received pelvic intensity-modulated radiotherapy (DT46Gy/23F, DT200cGy/F) and intracavitary brachytherapy (DT30Gy/5F, DT600cGy/F). Consolidation chemotherapy with the TP regimen (paclitaxel 175 mg/m^2^ and nedaplatin 75 mg/m^2^) was then administered for four cycles.

On 26 March 2021, the patient experienced a sudden episode of massive vaginal bleeding. She was readmitted due to massive vaginal bleeding, and subsequent imaging disclosed a cervical mass involving the lower uterine segment, with vesicovaginal and rectovaginal fistulas. The bilateral iliac lymph nodes had partially enlarged compared with previous scans, and bilateral hydronephrosis was noted, restaging the disease as stage IVA. From 13 May to 21 June 2021, the regimen was switched to a combination of albumin-bound paclitaxel (260 mg/m^2^) plus cisplatin (50 mg/m^2^), bevacizumab (15 mg/kg), and tislelizumab (200 mg) administered for two cycles. After two cycles, imaging assessment demonstrated SD. From 15 July 2021 to 12 April 2022, the patient received bevacizumab plus tislelizumab for 10 cycles. Imaging after two cycles of treatment showed a PR (**Figure 2**). Thereafter, due to financial constraints, tislelizumab was withheld from May to September 2022, during which she received only bevacizumab as maintenance therapy for five cycles. Notably, her hydronephrosis, which was first noted in April 2021 and initially managed conservatively without ureteral stenting, eventually required percutaneous nephrostomy on 11 March 2022.

**Figure 2:**
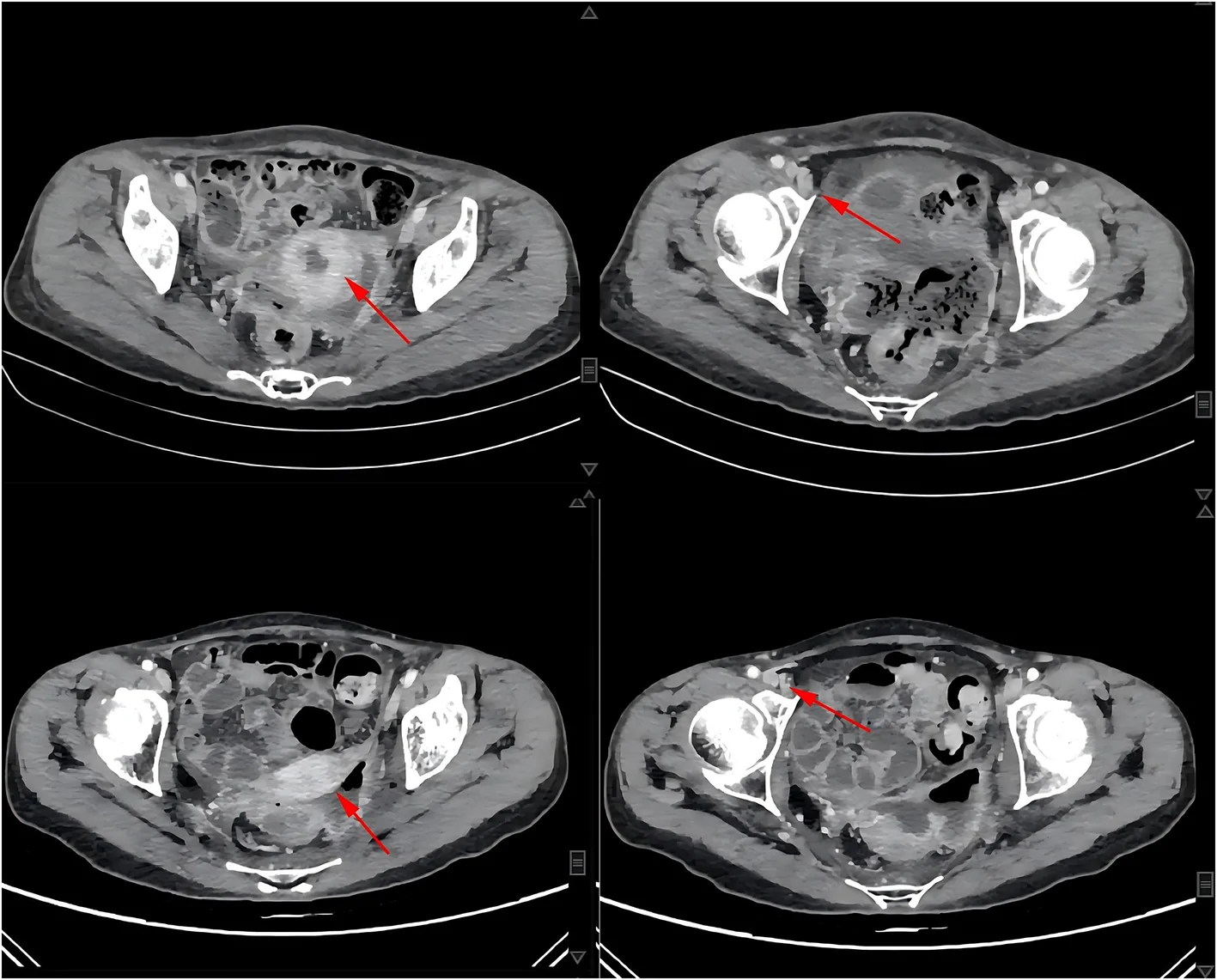
**(A)** Baseline contrast-enhanced CT scan of the pelvis demonstrating marked thickening of the rectal wall and pelvic fascia, with evidence of a rectovaginal–vesical fistula. **(B)** Baseline CT scan at a lower pelvic level revealing enlarged bilateral iliac lymph nodes. **(C)** Follow-up CT scan at the corresponding level to **(A)**, obtained after treatment, showing a marked reduction in rectal wall thickening and pelvic fascial thickening. **(D)** Follow-up CT scan at the corresponding level to **(B)**, acquired at the same time as **(C)**, demonstrating significant shrinkage of the bilateral iliac lymph nodes. Red arrows indicate the thickened rectal wall, fistulous tract, and enlarged lymph nodes.

However, imaging on 18 October 2022 revealed clear disease progression: the rectovaginal–pelvic–bladder fistula had enlarged, and both rectal and vaginal wall thickening had increased relative to earlier scans. Accordingly, PFS1—measured from the start of salvage therapy (April 2021) to this radiographic progression—was approximately 17 months.

From 25 October to 26 December 2022, the patient received three cycles of pemetrexed plus cisplatin plus a programmed cell death protein 1 (PD-1) inhibitor as a three-drug combination regimen. Imaging assessment afterward showed SD. On 7 June 2023, imaging showed that the extent of the rectovaginal–pelvic–vesical fistula had increased compared with the previous result and that the intestinal wall thickening was more pronounced than before, confirming further progression. PFS2—measured from the initiation of the second salvage regimen on 25 October 2022 to radiographic progression on 7 June 2023—was approximately 7.5 months. The OS from onset of the disease until death in September 2023 was approximately 43 months.

### 2.3 Case 3

The patient is a 57-year-old woman who was diagnosed with stage IB2 moderately differentiated cervical squamous cell carcinoma on 19 August 2019. Subsequently, the patient underwent concurrent chemoradiotherapy at another hospital, followed by four cycles of TP consolidation chemotherapy. In July 2021, the patient presented with right-sided flank pain, and subsequent imaging revealed multiple enlarged lymph nodes in the lower abdominal retroperitoneum and bilateral iliac regions, some of which were adherent to the left ureter and caused left hydronephrosis, which was staged as IV. At the same time, she had an ECOG PS of 2. From 2 August to 6 September 2021, she received a combination regimen of albumin-bound paclitaxel (260 mg/m^2^) plus nedaplatin (75 mg/m^2^), bevacizumab (15 mg/kg), and toripalimab (240 mg) for two cycles. Imaging after two cycles of treatment showed a PR. From 30 September 2021 to April 2022, she continued on bevacizumab plus a PD-1 inhibitor for six cycles. Imaging after two cycles of treatment showed a PR (**Figure 3**). Starting in May 2022, due to financial constraints, toripalimab was administered intermittently, and she continued on bevacizumab alone.

**Figure 3:**
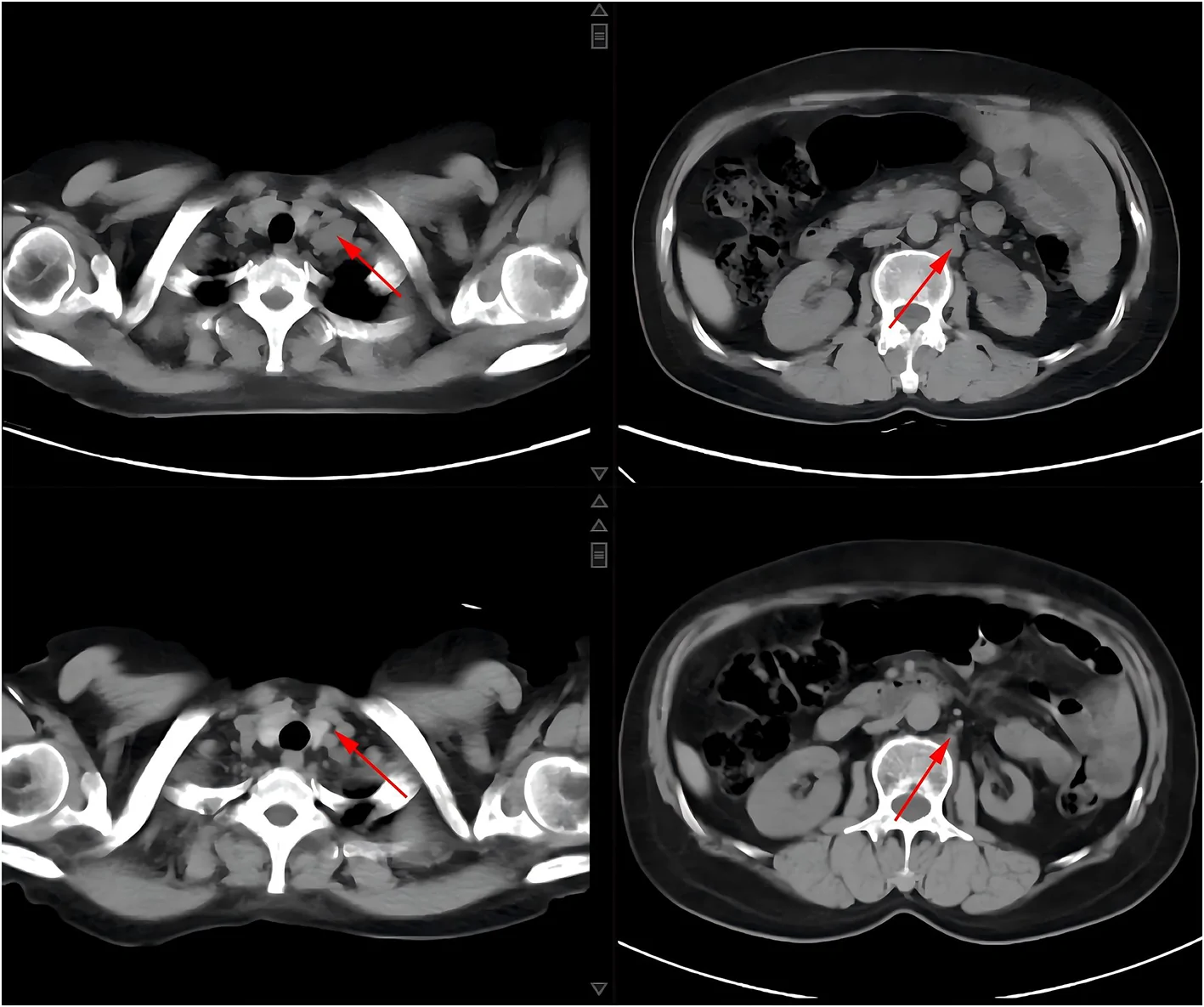
**(A)** Baseline contrast-enhanced CT scan of the neck and chest demonstrating multiple enlarged left supraclavicular lymph nodes. **(B)** Baseline CT scan of the abdomen and pelvis revealing multiple enlarged retroperitoneal and bilateral iliac lymph nodes. **(C)** Follow-up CT scan at the corresponding level to **(A)**, after therapy, showing partial regression of the left supraclavicular lymph nodes. **(D)** Follow-up CT scan at the corresponding level to **(B)**, acquired at the same time as **(C)**, showing partial reduction in size of the retroperitoneal and iliac lymph nodes. Red arrows indicate the representative metastatic lymph nodes.

On 12 June 2025, biopsy of a left axillary lymph node confirmed metastatic squamous cell carcinoma, marking the first disease progression. PFS1—from the start of initial salvage therapy on 2 August 2021 to this biopsy-confirmed progression—was 46 months. From 20 August to 22 December 2025, the combination of albumin-bound paclitaxel, nedaplatin, bevacizumab, and a PD-1 inhibitor was reinitiated for four cycles. From 2 February to 24 March 2026, she received bevacizumab plus a PD-1 inhibitor for two cycles. Imaging after two cycles of treatment showed a PR based on RECIST 1.1. PFS2 from the start of the second salvage therapy on 20 August 2025 to the last follow-up has exceeded 10 months. The OS from the initial diagnosis in August 2019 to the last follow-up in July 2026 has exceeded 83 months, and the patient remains under active follow-up.

### 2.4 Case 4

The patient is a 59-year-old woman who was initially diagnosed with cervical squamous cell carcinoma at another hospital in May 2019. Subsequently, she underwent 28 sessions of radiation therapy and four cycles of chemotherapy. As her initial imaging and full medical records were unavailable, we did not have data of the FIGO stage at diagnosis, along with the exact treatment details and doses. In January 2020, the patient came to our hospital for treatment and completed four sessions of intracavitary brachytherapy (300 cGy per session).

In December 2020, the patient presented with hematochezia and lower abdominal pain and was diagnosed with rectovaginal fistula. In March 2021, PET-CT revealed irregular thickening of the vaginal cuff and multiple enlarged lymph nodes along the bilateral internal iliac vessels, iliac fossae, and in the bilateral inguinal regions, with increased fluorodeoxyglucose (FDG) uptake. The recurrence was staged as IVA according to the FIGO 2018 classification.

Subsequently, she underwent surgery for the rectovaginal fistula. On admission in August 2021, she had an ECOG PS of 3, with signs of cancer cachexia. On 2 August and 27 August 2021, she received two cycles of the combination regimen of albumin-bound paclitaxel (80% of the standard 260 mg/m^2^) plus cisplatin (80% of the standard 50 mg/m^2^), bevacizumab (15 mg/kg), and toripalimab (240 mg). However, due to grade 4 myelosuppression (based on the Common Terminology Criteria for Adverse Events version 5.0), the triplet therapy was discontinued from the third cycle onward. After receiving supportive care including granulocyte colony-stimulating factor and platelet transfusions, her hematological parameters recovered and her cancer cachexia gradually improved. Imaging after two cycles of treatment showed a SD. On 20 September and 20 October 2021, the regimen was switched to maintenance therapy with bevacizumab plus toripalimab for two administrations. Subsequent imaging assessment showed SD. Her overall condition improved significantly, and she continued the dual-maintenance therapy at the local hospital. From 2024 onward, the treatment interval was gradually extended to once every 2–3 months with bevacizumab plus toripalimab. To date, no disease progression has been documented. From the start of salvage therapy to the last follow-up, PFS has exceeded 58 months. Up to now, the OS is 86 months, and the patient remains under active follow-up.

## 3 Discussion

### 3.1 Case 1

During the first cervical cancer recurrence, the patient developed peritoneal metastasis following surgery and chemoradiotherapy. A finding worth noting in this case is that, after receiving four cycles of induction therapy and five cycles of maintenance therapy for peritoneal metastasis, the patient had negative ascites cytology for malignant cells, but eventually relapsed. This finding warrants further interpretation. Firstly, the sensitivity of ascites cytology is limited, with false-negative rates reported to be as high as 30%–37% in the literature (10, 11). Secondly, a negative result merely indicates that no identifiable malignant cells were detected in the peritoneal fluid, but does not exclude the presence of microscopic residual disease on the peritoneal surface, tumor cell nests encased by fibrosis, or subperitoneal infiltration. Perhaps, in the near future, circulating tumor DNA (ctDNA) or circulating tumor cells (CTCs) may help predict treatment outcomes in cancer.

### 3.2 Case 4

Two aspects are noteworthy for this case. Firstly, although the patient had severe cachexia, an episode of grade 4 myelosuppression (which was effectively managed with supportive care), and an ECOG PS of 3, making her unsuitable for triplet induction therapy, she tolerated the subsequent dual-maintenance phase well. This suggests that such a combination regimen may also be feasible even for such patients, provided that close hematological monitoring is in place. In addition, after just two cycles of triple therapy followed by maintenance therapy, the patient has now been in remission for more than 5 years—a clinical pattern consistent with immune-mediated “trigger and sustain” effects.

We observed that all four patients derived varying degrees of clinical benefit from this individualized sequential approach. However, several aspects of the MIMT regimen still merit discussion. Firstly, the purpose of switching from triplet induction to dual maintenance is to discontinue cytotoxic chemotherapy. Compared with the conventional six-cycle regimen, two to four cycles may reduce the accumulation of chemotherapy-related toxicity. Secondly, the number of induction cycles was primarily determined by a combination of treatment response and patient tolerance. When the imaging findings after two to four cycles demonstrated disease control (i.e., SD or response according to RECIST 1.1), discontinuation of chemotherapy was considered on an individual basis, particularly when continued chemotherapy was associated with cumulative toxicity or was not considered likely to provide additional clinical benefit. For example, in case 3, we discontinued chemotherapy after only two cycles because a favorable response was observed. In case 4, chemotherapy was also stopped after two cycles, but for a different reason—grade 4 myelosuppression. In case 1, who tolerated the treatment well, we extended induction to four cycles. Finally, as this is a retrospective case series, these observations are intended to present our experience with these cases, and further validation in larger prospective studies is required.

However, several limitations of this case series warrant discussion. Firstly, for all four patients, baseline tumor tissue samples were not routinely tested for PD-L1 expression, mismatch repair (MMR) status, or tumor mutational burden (TMB) at the time of recurrence due to limited testing facilities and technical resources at our institution at that time. None of the four patients underwent these molecular tests. For one thing, we cannot exclude the possibility that any of the four carried MSI-H disease; if so, the impressive outcomes may be due more to a biologically favorable subset than to the broad utility of the MIMT. For another, PD-1 monotherapy typically achieves low response rates in proficient mismatch repair/microsatellite stable (pMMR/MSS) tumors (12). Nevertheless, all four of our patients experienced some clinical benefit. This raises an intriguing possibility: even pMMR/MSS patients may gain benefit from MIMT. This suggests that prospective studies incorporating these biomarkers may be required in the future to assess their impact on survival outcomes.

Secondly, none of the four patients completed the conventional six cycles of platinum-based chemotherapy, yet all derived meaningful benefit. This observation challenges the rigid reliance on a fixed number of cycles and raises the hypothesis that, in early responders, MIMT may offer comparable efficacy with reduced cumulative toxicity.

Thirdly, in this series, two patients (cases 2 and 4) had vesicovaginal and rectovaginal fistulas documented before the initiation of bevacizumab-containing therapy. In case 2, follow-up imaging revealed progressive enlargement of the fistulas during treatment. However, it is important to recognize that multiple confounding factors may have contributed to this worsening. Local tumor progression can directly invade the bladder and rectum, leading to fistula formation or exacerbation. In addition, prior pelvic radiotherapy—including intensity-modulated radiotherapy and brachytherapy—is a well-established risk factor for fistula development. Given these coexisting factors, a causal relationship between bevacizumab and fistula enlargement cannot be definitively established, although a contributory role cannot be entirely excluded. Extra caution should be exercised when administering bevacizumab to patients with fistulas.

Finally, this study lacked immunological monitoring data. As immunological parameters—such as peripheral blood lymphocyte subsets, cytokine levels, or PD-L1 staining on available tissue—were not routinely collected during treatment, we are unable to explain, from an immunological perspective, the biological basis of the observed durable disease control. This also suggests that future studies should incorporate dynamic monitoring of the immune profile in order to better investigate the potential mechanisms underlying disease control.

## 4 Patient perspective

Case3:“I have been living with this disease for over seven years, and I am still here with my family. This treatment gave me time I never expected to have. I am deeply grateful.”

Case4:“I couldn’t get out of bed or eat before treatment. Now I can do the things I love. I’m still here with my family, and that’s everything.”

## 5 Conclusions

This case series suggests that, in select patients with recurrent or metastatic cervical cancer, MIMT may be associated with disease control over the observed follow-up period. In these cases, transition to dual maintenance was undertaken after disease control was achieved, with the timing individualized according to treatment response and tolerability. We hope that this report may serve as a reference for clinical practice and provide insights for future prospective studies. However, given the small sample size of this case series, our findings require further validation in larger cohorts.

## Data Availability

The original contributions presented in the study are included in the article/**Supplementary Material**. Further inquiries can be directed to the corresponding authors.
