# Supplementary material for: Case Report: Prolonged survival with short-course triplet induction and dual maintenance in recurrent/metastatic cervical cancer
Source: Front Oncol. 2026 Oct 19;16:1961384. doi: 10.3389/fonc.2026.1961384 (PMC13724560; doi:10.3389/fonc.2026.1961384)
Supplement: Supplementary file 1 [file DataSheet1.pdf]

CARE case report guidelines

CARE Checklist of information to include when writing a case report

(cc) BY-NC-ND

Topic	Item	Checklist item description	Reported on Line
**Title**	1	The diagnosis or intervention of primary focus followed by the words "case report"	Title
**Key Words**	2	2 to 5 key words that identify diagnoses or interventions in this case report, including "case report"	Keywords section
**Abstract (no references)**	3a	Introduction: What is unique about this case and what does it add to the scientific literature?	Abstract
	3b	Main symptoms and/or important clinical findings	Case Presentation Abstract
	3c	The main diagnoses, therapeutic interventions, and outcomes	Abstract, Case Presentation, Table 1,Conclusions
	3d	Conclusion—What is the main "take-away" lesson(s) from this case?	Abstract, Conclusions
**Introduction**	4	One or two paragraphs summarizing why this case is unique (**may include references**)	Introduction
**Patient Information**	5a	De-identified patient specific information	Case Presentation
	5b	Primary concerns and symptoms of the patient	Case Presentation
	5c	Medical, family, and psycho-social history including relevant genetic information	Case Presentation, Discussion
	5d	Relevant past interventions with outcomes	Case Presentation, Table1
**Clinical Findings**	6	Describe significant physical examination (PE) and important clinical findings	Case Presentation, Table1
**Timeline**	7	Historical and current information from this episode of care organized as a timeline	Case Presentation, Table1
**Diagnostic**	8a	Diagnostic testing (such as PE, laboratory testing, imaging, surveys)	Case Presentation, Figure, Figure legends
**Assessment**	8b	Diagnostic challenges (such as access to testing, financial, or cultural)	Discussion
	8c	Diagnosis (including other diagnoses considered)	Case Presentation
	8d	Prognosis (such as staging in oncology) where applicable	Case Presentation, Discussion
**Therapeutic**	9a	Types of therapeutic intervention (such as pharmacologic, surgical, preventive, self-care)	Case Presentation,Table1
**Intervention**	9b	Administration of therapeutic intervention (such as dosage, strength, duration)	Case Presentation
	9c	Changes in therapeutic intervention (with rationale)	Case Presentation, Discussion
**Follow-up and**	10a	Clinician and patient-assessed outcomes (if available)	Case Presentation, Table1, Discussion
**Outcomes**	10b	Important follow-up diagnostic and other test results	Case Presentation, Figure, Figure

			legends
	10c	Intervention adherence and tolerability (How was this assessed?) ______	Case Presentation, Discussion
	10d	Adverse and unanticipated events ______	Case Presentation, Discussion
**Discussion**	11a	A scientific discussion of the strengths AND limitations associated with this case report ______	Discussion
	11b	Discussion of the relevant medical literature with references______	Introduction, Discussion, References
	11c	The scientific rationale for any conclusions (including assessment of possible causes) ______	Discussion, Conclusions
	11d	The primary "take-away" lessons of this case report (without references) in a one paragraph conclusion ______	Conclusions
**Patient Perspective**	12	The patient should share their perspective in one to two paragraphs on the treatment(s) they received ______	Patient Perspective
**Informed Consent**	13	Did the patient give informed consent? Please provide if requested ______	Informed Consent Statement<br>Yes☑ No☐
